# Urinary schistosomiasis in Nigeria: a 50 year review of prevalence, distribution and disease burden

**DOI:** 10.1051/parasite/2019020

**Published:** 2019-04-03

**Authors:** Charles Ogbonna Ezeh, Kenechukwu Chibuike Onyekwelu, Olaoluwa Phebian Akinwale, Lv Shan, Hu Wei

**Affiliations:** 1 Department of Medical Biochemistry, Faculty of Medical Sciences, University of Nigeria, Enugu Campus Enugu State Nigeria; 2 National Institute of Parasitic Diseases, Chinese Center for Disease Control and Prevention Shanghai PR China; 3 College of Life Science, Fudan University Shanghai PR China; 4 Nigerian Institute of Medical Research Lagos Nigeria

**Keywords:** Schistosomiasis, *Schistosoma haematobium*, Epidemiology, Snails, Morbidity, Praziquantel

## Abstract

We reviewed survey data deposited in the Global Neglected Tropical Diseases database and many other articles on the prevalence and distribution of *Schistosoma haematobium* in Nigeria. *Schistosoma haematobium* surveys conducted over the period of 50 years under review using different diagnostic tools revealed that Ogun State has the highest prevalence, followed by Ekiti state, while the lowest prevalence was recorded in Adamawa. No incidence of *Schistosoma haematobium* was recorded for states such as Akwa Ibom, Bayelsa, Nasarawa, Jigawa and Gombe. In terms of endemicity, this review has shown that Nigeria is divided into four zones: hyperendemic, moderately endemic, low endemic, and no endemic zones. A survey of 47 (15%) of the 323 dams in Nigeria revealed that 45 out of the 47 dams are located in the hyperendemic zone, while the remaining two are located in the moderately endemic zone. Twenty (43%) of the total surveyed dams harboured *Bulinus globosus* and/or *Biomphalaria pfeifferi*, the local intermediate hosts of schistosomes, and 18 of these are located in the hyperendemic zone, while the other two are in the moderately endemic zone. We conclude that there is an urgent need to carry out a nationwide survey to help in planning, coordinating, and evaluating schistosomiasis control activities.

## Abstract (fr)

Nous avons examiné les données recueillies dans la base de données mondiale sur les maladies tropicales négligées et de nombreux autres articles sur la prévalence et la répartition de *Schistosoma haematobium* au Nigéria. Les enquêtes sur *S. haematobium* menées au cours des 50 dernières années à l’aide de différents outils de diagnostic ont révélé que l’État d’Ogun avait la prévalence la plus élevée, suivi de l’état d’Ekiti, la plus faible prévalence ayant été enregistrée à Adamawa. Aucune incidence de *S. haematobium* n’a été enregistrée dans des États comme Akwa Ibom, Bayelsa, Nasarawa, Jigawa et Gombe. En termes d’endémicité, cette revue a montré que le Nigéria est divisé en quatre zones: hyper endémique, modérée, basse et sans endémie. L’enquête menée auprès de 47 (15 %) des 323 barrages au Nigeria a révélé que 45 des 47 barrages sont situés dans la zone d’hyperendémie, tandis que les 2 restants sont situés dans une zone d’endémie modérée. Parmi les barrages étudiés, 20 (43 %) hébergeaient *Bulinus globosus* et/ou *Biomphalaria pfeifferi*, les hôtes intermédiaires locaux des schistosomes, et 18 d’entre eux sont situés dans la zone d’hyperendémie tandis que les 2 autres se trouvent dans la zone d’endémie modérée. Nous concluons qu’il est urgent de mener une enquête à l’échelle nationale pour faciliter la planification, la coordination et l’évaluation des activités de lutte contre la schistosomiase.

## Introduction

Urinary schistosomiasis caused by *Schistosoma haematobium* is endemic in the sub-Saharan region of Africa, including in Nigeria [9, 10]. About 200 million people in some 74 countries are infected worldwide and at least 600 million are at risk of infection [27]. An estimated 120 million suffer severe consequences of the infection with an estimated annual mortality rate of about 20,000 worldwide [28]. An estimated 30 million Nigerians need to be treated annually for the disease [2]. In most endemic areas, the highest intensities of infection are found in children between 5 and 15 years of age [28]. In sub-Saharan Africa alone, it is estimated that 70 million individuals experience haematuria, 32 million difficulty in urinating (dysuria), 18 million bladder-wall pathology, and 10 million major hydronephrosis from infections caused by *S. haematobium* annually. The mortality rate due to non-functioning kidneys (from *S. haematobium*) and haematemesis has been estimated to be 150,000 per year [25]. The above figures show that urinary schistosomiasis is an important public health problem in sub-Saharan Africa, second only to malaria in morbidity [26]. Urinary schistosomiasis has been reported to increase the risk of HIV infection among women due to the fact that this parasite causes genital lesions and sandy patches [13]. Up to 75% of women infected with urinary schistosomiasis develop irreversible lesions in their vulva, vagina, cervix and uterus, creating a lasting entry point for HIV [20]. The planorbid snail *Bulinus* spp. is the intermediate host in the transmission of vesical schistosomiasis. The distribution of the disease is focal and its effects are more common in rural areas in the tropics where the population uses natural fresh water for their domestic water supply, recreational activities, and agricultural production. Hence, disease transmission is contingent on the presence of infected water, the primary snail host, and contact with the human population [17]. Extreme poverty, lack of knowledge of the risks, an inadequacy or total lack of public health facilities along with the unsanitary conditions in which millions of people live their daily lives, especially in the rural areas of developing tropical countries, are all factors contributing to the risk of infection [15, 29].

Although there is no current estimate for the disease in Nigeria, past estimates have calculated infection rates of about 25 million people and 101 million at risk of infection [7]. In terms of urinary schistosomiasis endemicity, Nigeria has been divided into three zones: a hyperendemic zone, a moderately endemic zone, and a zone with low or no endemicity [8].

Our review followed a very encompassing approach, covering half a century (1961–2011) of urinary schistosomiasis in Nigeria. Topics of interest were prevalence, disease burden, risk factors, effects of global health policies in terms of World Health Organization (WHO) programmes on schistosomiasis control and the federal government’s approach to disease surveillance, control of transmission, and the road map to morbidity control and elimination. Special reference was made to the effects of dams, lakes and other water bodies in Nigeria impacting the spatial and temporal distribution of *S. haematobium* and the future success of disease control programmes. The Nigerian government’s efforts towards morbidity control through primary healthcare policies over the last 50 years in line with the WHO’s eradication road map were highlighted.

The presence of two forms of human schistosomiasis, caused either by *Schistosoma haematobium* or by *Schistosoma mansoni*, in Nigeria has been known since 1881. A report by the WHO in 1987 indicated that the urinary form of the disease (caused by *S. haematobium*) is widespread throughout Nigeria, while intestinal schistosomiasis (caused by *S. mansoni*) is less prevalent and was not reported in the south-eastern and some south-western parts of Nigeria [1]. As a result, this review is restricted to *S. haematobium*.

## Methods

**Figure 1:**
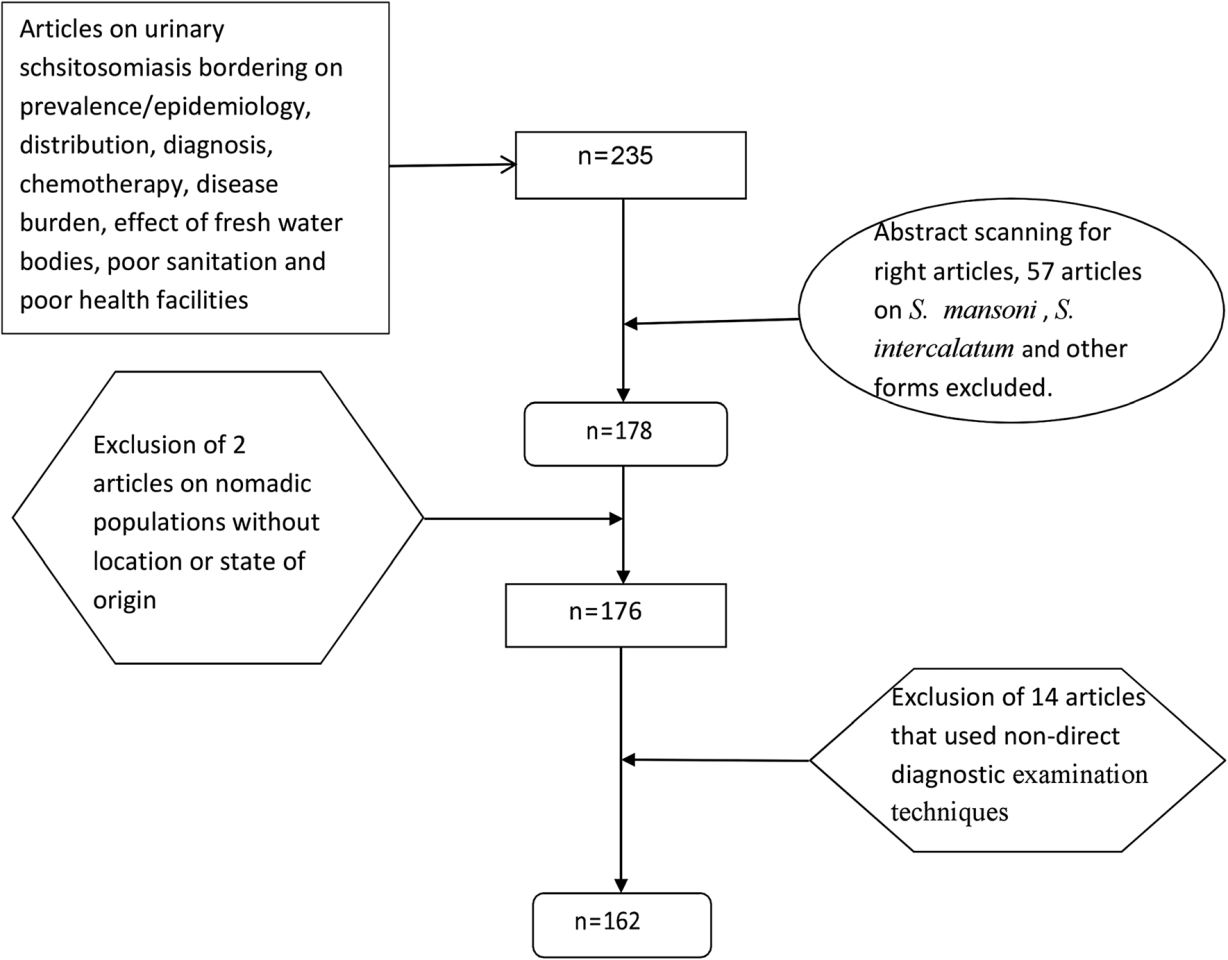
Article selection flow chart.

This review adapted in parts the methods of Hotez and Kamath [11] (2009) and the modified version of Schur et al. [21] (2011) as a framework outlined in the following steps (Fig. 1).

### Location, screening and selection of relevant publications

The literature search was done using an online literature database from 1961 to 2011, with urinary schistosomiasis listed as a neglected tropical disease on the PLoS Neglected Tropical Disease Website (http://www.gntd.org). This database collates general information about the type of publication, authors, and publication year, as well as study-specific information about the survey population, survey period, schistosome species, diagnostic test employed, and the number of infected individuals among those examined, stratified by age and sex (if available). We also examined all the journals related to *S. haematobium* epidemiology listed on African Journals Online (AJOL). The reference lists of identified articles and reviews were also searched manually, as were databases from the WHO, including the Weekly Epidemiological Record. All other publications on urinary schistosomiasis outside the mentioned databases were also included if they met the inclusion criteria.

### Publications selection/inclusion criteria

The review focused on 50 years of Nigeria’s existence from 1961, exactly a year after Nigeria gained independence to the last date in 2011 and as such, all articles on urinary schistosomiasis concerning prevalence/epidemiology, distribution, diagnosis, chemotherapy, disease burden, effect of fresh water bodies, poor sanitation and poor health facilities within the set dates were included. The search procedure yielded 235 articles. However, abstracts were scanned to determine whether or not the articles were valid and all independent articles on other species of schistosomes such as *S. mansoni* and *S. intercalatum* were excluded, giving 178 articles. Moreover, all articles on nomadic populations without the exact state of origin within the Nigerian Federation were excluded, as well as articles with conflicting figures. This left a total of 176 articles. Articles reporting non-direct diagnostic examination techniques, such as immunofluorescence tests and antigen detections, or publications reporting on questionnaire data alone were excluded, and these final exclusion criteria yielded 162 articles deemed relevant.

### Data extraction

The following information was extracted and recorded: general information about the type of publication, authors, publication year, as well as study-specific information about the survey population, the survey period, Schistosoma species (those other than *S. haematobium* were excluded), the diagnostic test employed, and the number of infected individuals among those examined stratified by age and sex (if available). These data formed the basis of the analysis.

### Study areas

The studied areas where surveys and sample collections were carried out within Nigeria were recorded as geographical coordinates in latitudes and longitudes. Almost all articles showed the coordinates of their survey points or delineated them in area maps. However, some survey point geographical coordinates were not provided at the year of study or publication. Therefore Google Earth map link tele-atlas, 2012 was used to obtain their coordinates. Nonetheless, there are cases of interstate surveys that cannot be represented in clear coordinates, but the prevalence data for each state were used.

### Sample collection

All articles indicated the time of urine sample collection as between 10 a.m. and 2 p.m. This is the time of midstream urine when all *S. haematobium* eggs shedding from patients were highest. All community-based surveys ensured pre-survey education of their volunteers before sample collection, while school-based surveys relied on school administrators to ensure accurate sample collection. Hospital-based survey used patients’ urine that presented haematuria/the eggs in diagnosis. All survey types took the precaution of ruling out menstruating female volunteers. Hence, all sample collections were reported to have followed standard urine sample collection procedures and conditions, and ensured the quality of the sample container. Ethical considerations were reported in each case, and ethical clearance was obtained from appropriate authorities.

### Urine analysis

The urine samples were reported to be analysed almost immediately after sample collections, except in a few cases where the survey points were very far from points of analysis. However, reports indicated that adequate provisions were made beforehand to forestall the time lag before analysis in terms of urine sample preservation. This was done by transporting the specimen in a cold box containing ice blocks to laboratories where they were analysed. Some articles reported immediate use of reagent strips in the field to determine haematuria and subsequent sedimentation/centrifugation or filtration before microscopy. There were many cases of negative haematuria with positive egg results in many urine samples. The infection intensity was defined as the number of *S. haematobium* eggs contained in a 10 mL urine sample and *S. haematobium* eggs were identified as having a terminal spine in microscopy.

## Results

### Prevalence and geographical distribution

**Figure 2:**
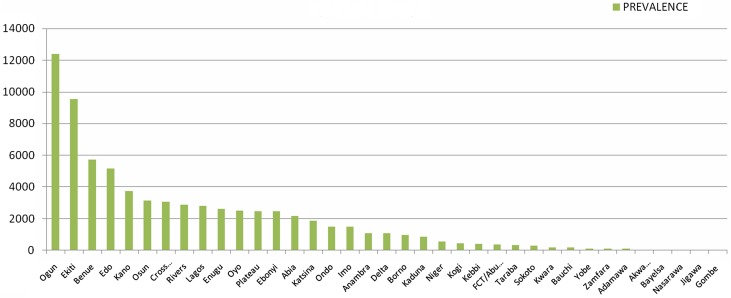
Summary of the prevalence of *Schistosoma haematobium* across the 36 states of Nigeria including FCT.

**Figure 3:**
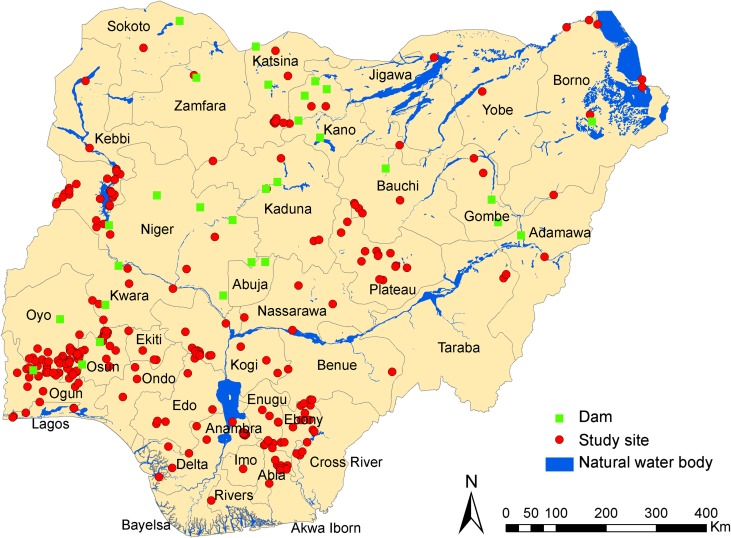
Map of Nigeria, showing location of study sites, dams, and natural water bodies.

**Figure 4:**
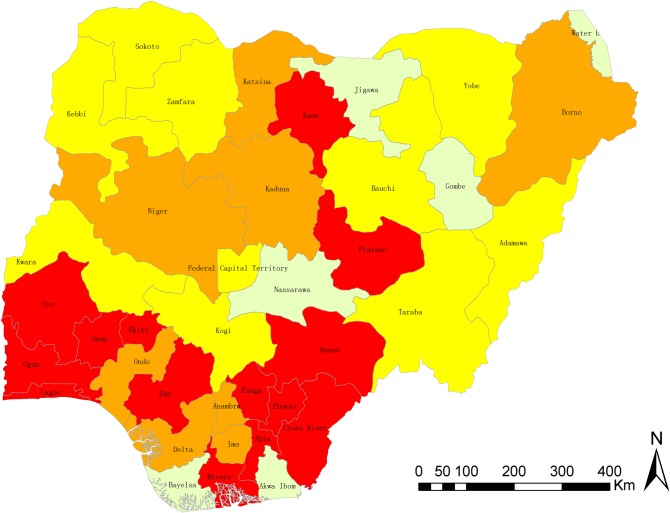
Map of Nigeria, showing the level of endemicity of urinary schistosomiasis by state. Red: highly endemic (>50%); brown: moderately endemic (10–50%); yellow: low endemic (1–10%); grey: no report of urinary schistosomiasis.

The exact degree of prevalence of urinary schistosomiasis in Nigeria is not known. The distribution and the prevalence level of the survey locations are shown in Figures 2–3 as well as Table 1, while ranking of the endemicity by state is shown in Table 2. An overview of the number of surveys with details given regarding sampling period, diagnostic technique, survey type, and prevalence, stratified by state is given in Table 1. Figure 2 is the bar chart representation of disease prevalence per state in Nigeria. Figure 3 is a map of Nigeria showing prevalence (cases per 100,000 inhabitants) and distribution of urinary schistosomiasis, while Figure 4 is a map of Nigeria showing the levels of endemicity of *S. haematobium*. These figures and tables are simply cumulative representations of various research work’s output included in this review. There are no baseline data or nationwide surveys carried out to serve as a reference point. It is known that states with low prevalence or without reports may be highly endemic with either *S. mansoni* or *S. intercalatum,* or have high prevalence of *S. haematobium* that is unreported. There are generally very few research reports on schistosomiasis in Nigeria. If we factor figures in Chitsulo et al. [7] (2000) and those at risk of infection and re-infection since the publication of the article into the prevalence, then Figure 3 will show a near real description of the geographical distribution of urinary schistosomiasis in Nigeria.

**Table 1:** Overview of the survey data included in the analysis stratified by state.

	Location		Survey year					Diagnostic technique				Survey type			Prevalence
	Total	Unique	1960s	1970s	1980s	1990s	2000+	RS	SD	FT	DT	CB	SB	HB	
Ogun	45	41	0	1	0	5	9	4	9	1	1	8	7	0	12,400
Ekiti	18	12	0	0	0	0	4	1	0	0	0	1	0	0	9464
Benue	19	9	0	0	1	0	4	0	4	1	0	3	1	1	5735
Edo	50	10	0	0	0	1	8	1	8	0	0	7	2	0	5174
Kano	7	6	0	0	1	0	3	1	3	0	0	2	2	0	3731
Cross River	11	1	0	0	0	5	7	1	4	7	0	3	8	1	3168
Osun	21	20	0	1	2	2	7	2	5	5	0	4	7	1	3138
Rivers	5	4	0	0	0	1	3	1	3	0	0	3	1	0	2868
Lagos	15	10	2	2	5	0	2	3	4	3	1	3	8	0	2809
Enugu	22	17	0	0	1	2	3	2	1	3	0	2	4	0	2626
Oyo	18	15	2	5	0	3	6	1	14	1	0	7	8	1	2517
Plateau	14	10	0	0	0	2	4	1	5	0	0	4	2	0	2476
Ebonyi	40	39	0	0	0	0	9	2	6	1	0	3	5	1	2471
Abia	31	23	0	0	1	2	2	1	3	1	0	3	2	0	2162
Katsina	6	4	0	5	0	0	0	0	4	1	0	3	2	0	1863
Ondo	19	18	0	0	0	0	2	0	2	0	0	2	0	0	1508
Imo	28	22	0	0	0	0	4	2	3	0	0	4	0	0	1485
Anambra	8	6	0	0	0	3	3	0	4	2	1	2	4	0	1100
Delta	41	16	0	0	0	0	2	0	2	0	0	0	2	0	1085
Borno	3	2	0	0	1	0	2	1	3	0	0	0	2	0	983
Kaduna	9	5	0	1	2	1	3	1	5	1	0	3	2	3	868
Niger	13	8	0	0	1	0	3	1	4	0	0	1	3	0	543
Kogi	22	18	1	0	0	0	1	1	2	0	0	1	1	0	450
Kebbi	1	1	0	0	0	1	0	0	1	0	0	1	0	0	406
FCT	6	5	0	0	0	0	1	0	1	0	0	1	0	0	360
Taraba	3	2	0	0	0	0	2	0	2	0	0	1	1	0	327
Sokoto	5	3	0	0	0	1	1	0	1	1	0	1	0	1	296
Kwara	1	1	0	0	0	1	0	0	1	0	0	0	1	0	193
Bauchi	4	2	0	0	0	1	0	1	0	0	0	0	1	0	181
Yobe	2	1	0	0	0	0	0	0	2	0	0	1	0	1	127
Zamfara	6	3	0	0	0	0	1	0	1	0	0	0	1	0	117
Adamawa	1	0	0	0	0	1	0	0	1	0	0	1	0	0	101
Akwa Ibom	–	–	–	–	–	–	–	–	–	–	–	–	–	–	–
Bayelsa	–	–	–	–	–	–	–	–	–	–	–	–	–	–	–
Nasarawa	–	–	–	–	–	–	–	–	–	–	–	–	–	–	–
Jigawa	–	–	–	–	–	–	–	–	–	–	–	–	–	–	–
Gombe	–	–	–	–	–	–	–	–	–	–	–	–	–	–	–

**Table 2:** Ranking of endemicity by state.

Endemicity	States
Hyperendemic zone (>50%)	Abia, Ebonyi, Enugu,Osun, Ogun, Lagos, Rivers, Cross Rivers, Edo, Benue, Kano, Oyo, Ekiti, Plateau
Moderately endemic zone (10%–50%)	Anambra, Ondo, Imo, Delta, Borno, Kaduna, Niger, Katsina
Low endemic zone (1%–10%)	Kogi, Taraba, Adamawa, Kebbi, Sokoto, Bauchi, Yobe, Kwara, Zamfara, Abuja
No report	Gombe, Akwa Ibom, Jigawa, Nasarawa, Bayelsa.

**Table 3:** The distribution of the snail host and human schistosomiasis in investigated artificial lakes/dams in Nigeria.

S/N	Name of dam	Location	Size	Purpose	Year	Snail	Human infection
1	Kubani	Kaduna	Medium	WS	1975	Positive	NI
2	Kangimi	Kaduna	Large	WS, IR	1977	Positive	NI
3	Bagoma	Kaduna	Large	WS, IR	1974	Positive	NI
4	Zaria	Kaduna	Large	MP	1974	Positive	NI
5	Baugauda	Kano	Large	MP	1970	Positive	NI
6	Karaye	Kano	Large	MP	1971	Negative	NI
7	Tiga	Kano	Large	MP	1975	Positive	Positive
8	Jakara	Kano	Large	MP	1976	Negative	NI
9	Ruwan Kanya	Kano	Large	MP	1976	Positive	NI
10	Tomas	Kano	Large	MP	1976	Positive	Positive
11	Kafin Chiri	Kano	Large	MP	1977	Negative	NI
12	Tudun Wada	Kano	Large	MP	1977	Negative	NI
13	Gari	Kano	Large	MP	1980	Negative	NI
14	Marashi	Kano	Large	MP	1980	Negative	NI
15	Pada	Kano	Large	MP	1980	Negative	NI
16	Watari	Kano	Large	MP	1980	Negative	NI
17	Magaga	Kano	Large	MP	1990	Positive	NI
18	Challawa Gorge	Kano	Large	MP	1992	Negative	NI
19	Kango	Kano	Large	MP	UC	Negative	NI
20	Warwade	Kano	Large	MP	NA	Negative	Negative
21	Birmin Kudu	Kano	Small	MP	1970	Negative	NI
22	Rimin Gado	Kano	Small	WS,RC	1978	Positive	Positive
23	Dogwala	Kano	Small	FL	NA	Negative	NI
24	Duduvum	Kano	Small	FL	NA	Negative	NI
25	Gata	Kano	Small	FL	NA	Negative	NI
26	Garanga	Kano	Small	FL	NA	Negative	NI
27	Rugunsana	Kano	Small	FL	NA	Negative	NI
28	Gulka	Kano	Small	FL	NA	Negative	NI
29	Iggi	Kano	Small	FL	NA	Negative	NI
30	Guzu Guzu	Kano	Small	FL	NA	Negative	NI
31	Kefin Gana	Kano	Small	FL	NA	Negative	NI
32	Kiwia	Kano	Small	FL	NA	Negative	NI
33	Kiyako	Kano	Small	FL	NA	Negative	NI
34	Kara Dumba	Kano	Small	FL	NA	Negative	NI
35	Malumfashi	Katisina	Large	MP	NA	Positive	Positive
36	Zobe	Katisina	Large	MP	NA	Positive	Positive
37	Kainji	Niger	Large	MP	1968	Positive	Positive
38	Oyan	Ogun	Large	MP	NA	Positive	Positive
39	Opa	Osun	Large	WS	1980	Positive	Positive
40	Eleiyele	Oyo	Large	WS	1942	Positive	NI
41	Oba	Oyo	Large	WS	1964	Positive	NI
42	Opeki	Oyo	Large	WS	1967	Positive	NI
43	Wurno	Sokoto	Small	MP	1960	Positive	Positive
44	Goronyo	Sokoto	Large	MP	1983	Negative	Negative
45	Bakolori	Zamfara	Large	MV	1982	Positive	Positive

Notes: MP = Multipurpose use, WS = Water supply, RC = Recreation, IR = Irrigation, NA = Not available, NI = Not investigated, FL = Flood control, UC = Under construction, S/N = Serial number, Nil = Not detected.

Large dams are all dams >15 m high or all dams with height 10–15 m having length of crest >500 m and/or reservoir capacity >1 million m^3^. Medium dams are all dams with height 8–10 m; and Small dams are all dams <8 m high.

### Disease burden

Nigeria ranks highest in terms of schistosomiasis burden among the countries in sub-Saharan Africa (SSA) [11]. Of the 192 million cases estimated in SSA by Steinmann et al. [22], Nigeria alone has 29 million cases. The disease burden, *i.e.,* Disability-Adjusted Life Year (DALYs) in sub-Saharan Africa resulting from schistosomiasis is estimated to be 1.6–4.2 million, which is 93% of estimated global disease burden in DALYs, put at 1.7–4.5 million [11]. Of this estimate, Nigeria ranks highest as of 2006. Urinary schistosomiasis accounts for more than 90% of all schistosomiasis cases in Nigeria, suggesting that the DALYs in Nigeria resulting from schistosomiasis are mainly from urinary schistosomiasis.

### Control measures

Currently, there is no separate control measure applicable in Nigeria other than the measures the Federal Ministry of Health outlined in its Primary Health Policy covering all infectious diseases. However, Nigeria has adopted the WHO’s strategy for schistosomiasis control but there is an apparent gap in policy making and implementation. Recently, the Carter Center-assisted programme in Nigeria provided health education and schistosomiasis treatment to communities in four states – Plateau, Delta, Edo, and Nasarawa – where the burden of the disease is high, and as of 2011, the programme has assisted by providing more than 6 million cumulative praziquantel tablets which have been distributed primarily to children since 1999. The vast majority of these treatments were distributed during 2008–2011 alone, through a scale up of activities made possible through a donation to the Center from the World Health Organization and Merck KGaA (Germany) (http://www.cartercenter.org/health/schistosomiasis/index.html). The remaining 33 states and Federal Capital Territory are meant to be covered under the Mass Drug Administration (MDA) programmes of the WHO and the Federal Ministry of Health.

## Discussion

There is currently no vaccine for schistosomiasis. Control measures rely on the use of a chemotherapeutic drug – praziquantel, which provides a safe and effective oral treatment against all human schistosome species. It is an essential tool that led to a shift in the global control strategy from transmission containment to morbidity control that occurred in the mid-1980s [19].

WHO has set a target to regularly treat 75% of school-aged children at risk of morbidity due to schistosomiasis by 2010, in support of the 54th World Health Assembly which passed a resolution urging member states to provide drug treatment to high-risk groups [6] and in 2002, a grant from the Bill and Melinda Gates Foundation (BMGF) to the Schistosomiasis Control Initiative (SCI), Imperial College of Science Technology and Medicine, enabled the initiation of country-wide control programmes in six African countries. The increasing expansion of international initiatives to reduce the disease burden of helminth infections in the developing world has been catalysed by “funding from the BMGF, donations from several drugs companies, and the reduced price of praziquantel” [14]. But how would all these measures fare in Nigeria considering that there is no clear road map for schistosomiasis control in the country, no accurate national data on prevalence and distribution, treatment and the predisposing factors to re-infection after treatment?

In Nigeria, *S. haematobium* infection had been found in many parts of the country with varying intensities and prevalence rates, and incidence is believed to be on the increase [19]. The true epidemiological data appear difficult to determine in developing nations because of inadequate research and no epidemiological control/information centre on tropical diseases, despite its relevance in planning for control in any locality. It is known that schistosomiasis affects up to 50% or more of people in some areas of Nigeria, but the total number of affected persons is unknown. Endemicity may be linked to behaviour, lack of education, public health facilities, very poor sanitary conditions and poverty in this part of the world [24]. The prevalence of this disease like many other endemic diseases is affected by the socio-cultural characteristics of Nigeria. As a result, the prevalence of this disease is also affected by these characteristics and accounts for the obvious difference in the distribution of the disease between southern and northern parts of the country [30]. Even within the same geographical area, such differences are likely to exist between different age groups. Although the prevalence of urinary schistosomiasis is found not to be uniform over the five decades in the states studied, the distribution among sexes was similar in that more males were infected than females, and this finding is statistically significant (*p* < 0.05) [4, 17, 23]. However, some studies in the southern parts of Nigeria showed that females have a higher prevalence of urinary schistosomiasis than males [3, 16]. The reason for this is that women are mostly involved in activities known to favour infection due to their gender-assigned responsibilities.

Socio-cultural factors like washing, fishing and recreational activities in fresh water harbouring infected snails facilitate the transmission of the disease. These practices are very common in the rural areas of northern Nigeria and many other villages in the western and eastern parts of the country. The construction of earth dams for dry season farming contributes to the high prevalence and distribution of the disease in this part of the country. Also, communities around most fresh water bodies show very high prevalence (Fig. 4). A total of 47 (15%) of the 323 dams in Nigeria have been surveyed for the presence of the snail intermediate host species of schistosomiasis, taking into consideration its distribution and ecology. The survey revealed that 45 of the 47 dams are located in the hyperendemic zone, while the remaining two are located in the moderately endemic zone. Results show that 20 (43%) of the total surveyed dams harboured *Bulinus globosus* and/or *Biomphalaria pfeifferi*, the local intermediate host of the Schistosoma parasite. Eighteen of these are located in the hyperendemic zone, while the other two are in the moderately endemic zone. Fifteen of them are in the northern part of the country and the remaining five are in the southwest [18]. Since the intermediate host of schistosomiasis breeds in slow-flowing/stagnant water, reservoirs of dams provide favourable conditions for year-round transmission of the disease, even in areas where snail distribution used to be seasonal [5]. Imevbore et al. [12] showed that within the reservoirs, distribution is focal and is confined to human contact sites, especially along their shallow vegetative shore, not more than a few meters from the shores or deep into the water. The endemicity ranking by state (Table 2) shows some areas of contrast in that some well-known states classified by Cowper et al, 1965 as hyperendemic are now in moderately endemic or low endemic zones. Obviously, this is due to low research levels or survey data over the decades in review. Hence, Table 2 cannot really serve as a reference as the data forming the classification represented in the table are grossly insufficient owing to very low research levels and survey data. The information is nonetheless informative. The merging of data arising from the prevalence of urinary schistosomiasis predicted due to dams and the data in Table 2 give a better estimated prevalence of the disease over the last 50 years so far reported in Nigeria within the context of this review inclusion criteria. Therefore, Figure 3 represents the total known cases of urinary schistosomiasis reported in various surveys over the five decades. Conclusively, it is very clear that the Federal Ministry of Health should urgently carry out a national survey to guide the various programmes for schistosomiasis control. More research is needed to elucidate the transmission patterns of different species and as well an overall political will on the part of government to cut transmission through several measures: mass education, provision of potable water, and proper waste disposal facilities to all rural areas (especially settlements around dams), and adequate checks on sanitary conditions of residents and a continuous MDA programme.

## Conclusion

We conclude that there is an urgent need to carry out a nationwide survey to help in planning, coordinating, and evaluating schistosomiasis control activities.

## Competing interests

The authors declare that they have no competing interests.
